# Significance of hepatitis B virus capsid dephosphorylation via polymerase

**DOI:** 10.1186/s12929-024-01022-9

**Published:** 2024-04-01

**Authors:** Chih-Hsu Chang, Chiaho Shih

**Affiliations:** 1 Institute of Biomedical Sciences, Academia Sinica, Taipei, 112 Taiwan; 2 Graduate Institute of Medicine, Kaohsiung Medical University, Kaohsiung, 807 Taiwan; 3 Graduate Institute of Cell Biology, China Medical University, Taichung, 406 Taiwan

**Keywords:** Hepatitis B virus (HBV), HBV core protein (HBc), Capsids dephosphorylation (de-P), Phosphatase, Polymerase (pol), RNase H domain

## Abstract

**Background:**

It is generally believed that hepatitis B virus (HBV) core protein (HBc) dephosphorylation (de-P) is important for viral DNA synthesis and virion secretion. HBV polymerase contains four domains for terminal protein, spacer, reverse transcriptase, and RNase H activities.

**Methods:**

HBV Polymerase mutants were transfected into HuH-7 cells and assayed for replication and HBc de-P by the Phos-tag gel analysis. Infection assay was performed by using a HepG2-NTCP-AS2 cell line.

**Results:**

Here, we show that a novel phosphatase activity responsible for HBc de-P can be mapped to the C-terminal domain of the polymerase overlapping with the RNase H domain. Surprisingly, while HBc de-P is crucial for viral infectivity, it is essential for neither viral DNA synthesis nor virion secretion. The potential origin, significance, and mechanism of this polymerase-associated phosphatase activity are discussed in the context of an electrostatic homeostasis model. The Phos-tag gel analysis revealed an intriguing pattern of “bipolar distribution” of phosphorylated HBc and a de-P HBc doublet.

**Conclusions:**

It remains unknown if such a polymerase-associated phosphatase activity can be found in other related biosystems. This polymerase-associated phosphatase activity could be a druggable target in clinical therapy for hepatitis B.

**Supplementary Information:**

The online version contains supplementary material available at 10.1186/s12929-024-01022-9.

## Background

Hepatitis B virus (HBV) is a human hepatotropic DNA virus (hepadnavirus) [5, 53, 63]. Chronic active hepatitis B patients have a higher risk to develop liver cirrhosis and hepatocellular carcinoma. At present, no treatment can effectively eradicate the virus in the liver reservoir of chronic patients [51]. Therefore, continuous lifetime treatment is required to inhibit HBV from reactivation. It is inevitable that long term treatment can lead to the emergence of drug resistant variants.

**Fig. 1:**
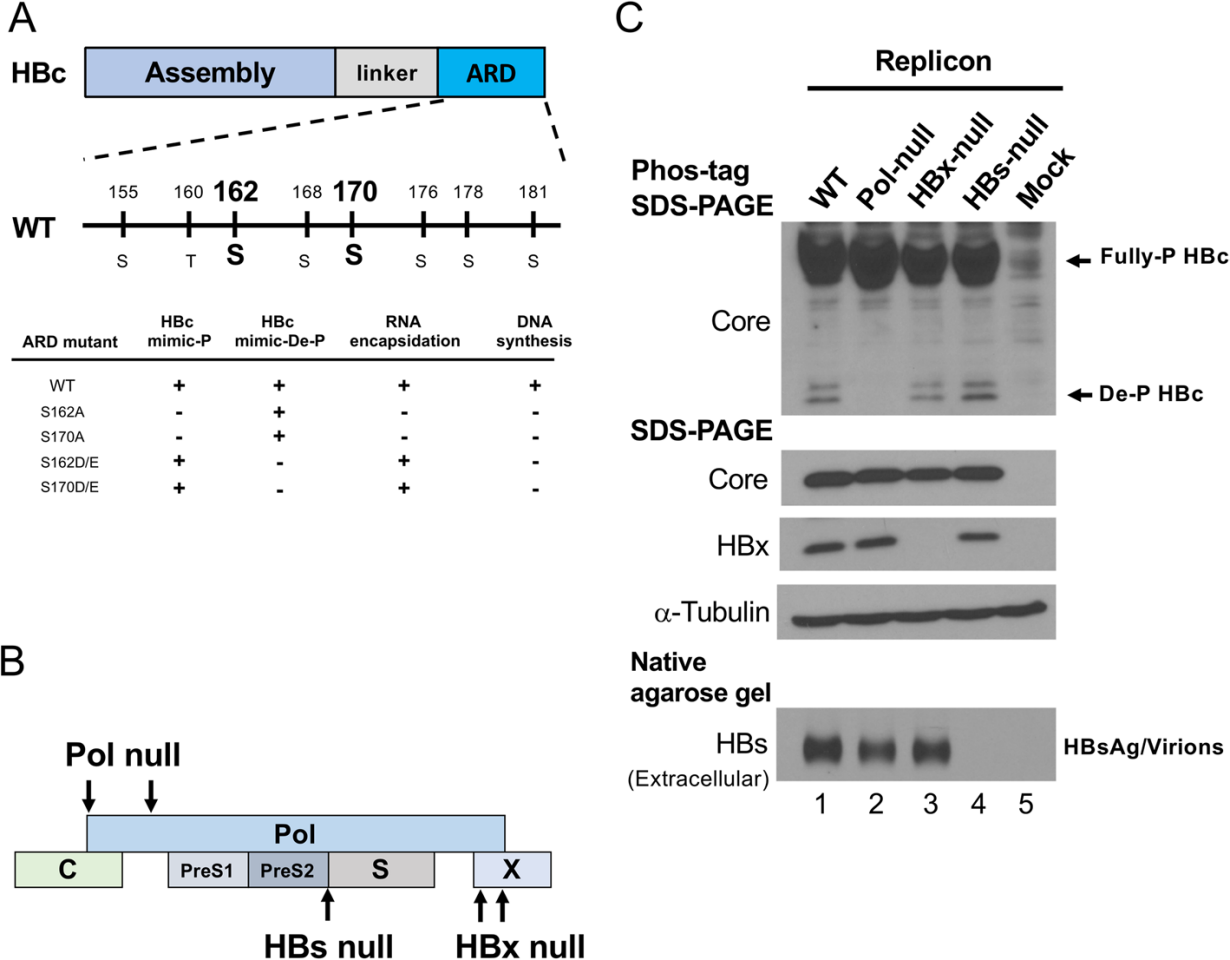
Dephosphorylation of HBV core protein (HBc) is correlated only with the presence of HBV polymerase, but not with the HBs envelope or the HBx protein.** A** The C-terminus of HBc contains three major and four minor phosphorylation sites. A summary of previous phosphomimicking mutagenesis studies on the major phosphoacceptor sites of HBc serine-162 and serine-170. Mutant S162D/E and mutant S170D/E enabled HBV pregenomic RNA encapsidation, but not viral DNA synthesis. **B** Cartoon illustration of three HBV mutant plasmids used in Fig. 1C. A mutant pol null is ablated at the first ATG initiation codon and the second ATG codon is engineered into a stop codon TAG. Mutant HBx null contains two engineered TAA stop codons in the HBx gene. Mutant HBs null lost the initiation codon of the small envelope protein. **C** Top panel: Phos-tag gel electrophoresis separated the fully phosphorylated HBc from HBc with partial or no phosphorylation. Completely dephosphorylated (De-P) HBc migrated faster than phosphorylated HBc. Different mutants in Fig. 1B produced similar amounts of HBc by Western blot analysis. Middle panel: Lack of HBx protein from mutant plasmid HBx null by Western blot using an anti-HBx antibody. Bottom panel: Mutant HBs null produced no HBs envelope protein in the medium by NAGE and Western blot using an anti-HBs antibody. Pol is known to be expressed at a very low level and no good antibody for pol is available

HBV core particles contain endogenous kinase activity and can be labelled with ^32^P isotope [18, 68]. It has been known for decades that phosphorylation and dephosphorylation (de-P) of HBV core protein (HBc) play an important role in HBV life cycle, including pregenomic RNA (pgRNA) encapsidation, capsid assembly, reverse transcription and virion secretion [4, 11, 16, 30, 32, 34, 39, 44, 56, 59, 69, 70, 74]. Lan et al. [32] and Gazina et al. [16] first performed phosphomimetic mutagenesis at three major serine (ser or S) phosphoacceptor sites at ser-155, ser-162 and ser-170 of HBc. Changing from serine to phosphomimicking aspartic acid (D) or glutamic acid (E) (S162D/E and S170D/E), supported HBV pgRNA encapsidation, but not viral DNA synthesis (Fig. 1A). In contrast, changing from serine to de-P-mimicking alanine (A) (S162A and S170A), resulted in the lack of pgRNA encapsidation, and thus no consequent pgRNA-templated DNA synthesis. These results suggested that while HBc serine phosphorylation is required for pgRNA encapsidation, it is inhibitory for viral DNA synthesis. These results have always been interpreted as implying, albeit with no direct experimental proof, that HBc de-P is required for viral DNA synthesis. Indeed, reversible and dynamic HBc (de)phosphorylation is considered an important regulatory mechanism in the hepadnaviral life cycle [4, 30, 69, 70, 74]. A recent study also compared HBV core (de)phosphorylation between a replicon and a non-replicon system, and a correlation between HBc de-P and viral replication was observed [56]. Many cellular kinases have been reported to be involved in HBc phosphorylation [12, 13, 19, 26], while few cellular phosphatases were proposed to be involved in HBc de-P [22, 65].

Another highly popular concept is that core de-P could facilitate, or is required, for HBV virion secretion [44, 52]. Using a biochemistry approach in a duck hepatitis B virus (DHBV) model, Pugh et al. [44] compared HBc phosphorylation between the intracellular capsids and secreted extracellular virions. Intracellular core protein is fully or partially phosphorylated, relative to the extracellular virion-associated core protein. Because both HBc ser-162 and ser-170 are important phosphorylation sites, mutants S162A and S170A are severely defective in pgRNA encapsidation and DNA synthesis [16, 32] (Fig. 1A). It is therefore impossible to know whether S-to-A core mutants (mimicking HBc de-P) are better or worse in the late event of genome-containing virion secretion than WT-DHBV or S-to-D/E mutants (mimicking HBc phosphorylation) [40].

In this study, we succeeded in dissociating the tight correlations between HBc de-P, viral DNA synthesis, and virion secretion. Surprisingly, several site-directed mutations in the RNase H domain of HBV polymerase abolished HBc de-P, yet spared the bulk activity of viral DNA synthesis, and exhibited only a minor reduction (less than 2-fold) in virion secretion. Core dephosphorylation is neither necessary nor sufficient for viral DNA synthesis and virion secretion. Strikingly, these RNase H domain mutants defective in HBc de-P displayed a 10-fold reduced viral infectivity in cell culture, despite the fact that they are replication competent in normal viral DNA synthesis in the plasmid DNA transfection system.

## Materials and methods

### Cells and transfection

HuH-7 cells, HepG2-NTCP-AS2 cells and HEK293T cells were maintained in DMEM medium (Invitrogen) supplemented with 10% fetal bovine serum (Gibco), 100 U/ml penicillin and 100 µg/ml streptomycin. HepG2 cells were transduced by the NTCP-expressing lentivirus, followed by selection with 40 µg/ml blasticidin. After serial dilutions and passages, a single cell clone of HepG2-NTCP-AS2 cells was isolated. Plasmid DNA were transfected by using PolyJet™ In Vitro DNA Transfection Reagent (Signagen).

### Anti-viral reagents

HBV RT inhibitors Lamivudine, Tenofovir, Clevudine, Emtricitabine and Entecavir were purchased from Selleck Chemicals. Myrcludex B (MyrB) is a synthetic lipopeptide of the HBV pre-S1 domain from MYR Pharmaceuticals.

### Antibodies

A rabbit anti-core antibody [36, 67] (1:2000) was used for Western blot analysis, Phos-tag SDS-PAGE, native agarose gel electrophoresis (NAGE) (1:5000), and immunofluorescence analysis (IFA) (1:200). A rabbit anti-HBx antibody (Abcam, ab39716) (1:2000) was used for Western blot analysis of HBx protein. A goat anti-HBs antibody (B0560, Dako) (1:5000) was used for detecting HBs by NAGE. A mouse α-tubulin antibody (DM1A) (Genetex, GTX11302) (1:2000) was used for Western blot analysis. A mouse HA-tag antibody (Genetex, GTX115044) (1:1000) was used for immunoprecipitation assay (2 µg) and Western blot analysis. A mouse anti-Flag M2 antibody (Sigma, F1804) (1:2000) was used for immunoprecipitation assay (2 µg) and Western blot analysis.

### Plasmids

An HBV replicon plasmid pCHT-9/3091 contains a 1.1mer HBV genome (genotype D, ayw) (30). The epsilon mutants and RNase H mutants V686E, V698D and L712E were derived from replicon pCHT-9/3091. Plasmid pMT-pol is a WT polymerase expression vector [45]. The Pol mutants and RNase H mutants were generated from pMT-pol for complementation with a pol-null pCHT-9/3091 replicon [6]. Site direct mutagenesis was generated by using a QuikChange Lightning Site-Directed Mutagenesis Kit (Agilent). The 3xHA-Pol expression plasmid was generated by cloning the polymerase ORF from pCHT9/3091 into pCDNA3.1, and a 3xHA epitope was inserted in-frame into a position upstream and adjacent to the ATG start site of Pol by site directed mutagenesis. The Flag-tagged PP1α, PP1β and PP1γ expression plasmids were purchased from Genecopoeia.

### Phos-tag SDS-PAGE

HBV transfected cells in six-well plates were harvested at 5 days post transfection. Capsids were purified through a 20% sucrose cushion before analysis on the polyacrylamide-Mn^2+^ bound Phos-tag SDS-PAGE (Wako Pure Chemical Industries, Richmond, VA, USA) [28, 38, 56]. The subsequent Western blot analysis was performed by using a rabbit anti-core antibody [6, 36, 67] (1:2000).

### Native agarose gel electrophoresis (NAGE)

Cell lysates and supernatant of HBV replicon transfected HuH7 cells were collected at 5 days post-transfection. Intracellular or extracellular HBV particles were resolved by 1% native agarose gel electrophoresis, followed by Western and Southern blot analyses [9, 56]. HBV capsids and HBsAg/virions were detected with a rabbit anti-core antibody (1:5000) and a goat anti-HBs antibody (B0560, Dako) (1:5000), respectively. The digoxygenin (DIG)-labeled full-length HBV specific DNA probe was used for hybridization in Southern blot analysis.

### HBV infection

HBV virions were collected from the supernatant of HBV replicon-transfected cell culture. For hepatocyte differentiation, HepG2-NTCP-AS2 cells were maintained in DMEM with 2.5% DMSO for 48 h. Virion-associated HBV DNA was estimated by substraction of the naked capsid-associated HBV DNA from the total viral DNA. Briefly, the amount of virion-associated HBV DNA was estimated by first measuring the ratio of Southern blot banding intensities between the slow-migrating virions (V) and the faster-migrating naked capsids (NC) on the native agarose gel electrophoresis (NAGE). This V/(V + NC) ratio from Southern blot was then used to calculate the amount of virion-associated HBV DNA (VGE), by multiplication of this ratio with the total amount of HBV DNA (V + NC) measured by qPCR [66]. Differentiated HepG2-NTCP-AS2 cells were infected with HBV for 16 h at 500 virion genome equivalent (VGE) per cell in DMEM medium containing 2% FBS, 4% PEG-8000 and 2.5% DMSO [8]. HBV infection was detected by immunofluorescence analysis (IFA) and ELISA. For the HBV attachment assay, HepG2-NTCP-AS2 cells were inoculated with 2000 HBV VGE/cell at 4˚C for 4 h in the PEG-free DMEM medium. After washing by 1X PBS, the cell surface-associated HBV DNA was measured by qPCR assay. For HBV internalization assay, HepG2-NTCP-AS2 cells were inoculum with 2000 VGE/cell of HBV at 4˚C for 4 h in PEG-free DMEM medium. After washing by 1X PBS, cells were further incubated at 37 ˚C for 24 h to allow viral internalization. The internalized HBV DNA was assayed by qPCR.

## Other experimental procedures

Preparations of capsid-associated RNA, DNA and total intracellular RNA were as described elsewhere [11, 34, 39, 56]. Immunofluorescence analysis (IFA) for HBc was performed as detailed previously [36, 67]. The images of HBc positive cells were scored by the MetaMorph analysis software (Molecular Devices). ELISA for HBsAg and HBeAg were according to the vendor’s protocol. Quantification of HBV DNA by qPCR was performed as reported elsewhere [6]. Co-immunoprecipitation and Western blot assay were performed as detailed elsewhere [6].

### Quantification and statistical analysis

All Statistical analyses were performed by the GraphPad Prism software. The statistical significance was analyzed by Student’s t test. ***P < 0.001; **P < 0.01; *P < 0.05.

## Results

### Phosphorylation status of HBV core protein

The arginine-rich domain (ARD) at the C-terminus of HBc contains three major and four minor phosphorylation sites, in addition to one non-phosphorylated serine-181 [12, 19] (Fig. 1A). Different degrees of HBc phosphorylation can be well resolved by using the so-called Phos-tag gel electrophoresis [19, 56]. For example, in Supplementary Figure S1, mutant 8A mimicked the completely dephosphorylated HBc by changing all the phosphorylation sites into an alanine. HBc of mutant 8A migrated much faster than the phosphorylated HBc on the Phos-tag gel.

### Pol is needed for HBc dephosphorylation

HBV is known to encode four major proteins [53]: the core/precore proteins (HBc), polymerase (pol), envelope proteins (preS1/preS2/S), and the HBx protein (Fig. 1B). We asked whether any of these viral proteins could influence core protein (de)phosphorylation. As shown in Fig. 1C, we ablated each viral protein individually in the replicon plasmid, followed by plasmid transfection into HuH-7 cells for the Phos-tag gel assay. Neither the envelope HBs protein nor the HBx protein is required for HBc de-P. Only the absence of the polymerase resulted in the loss of HBc de-P.

### The RNA packaging signal epsilon (ε)

**Fig. 2:**
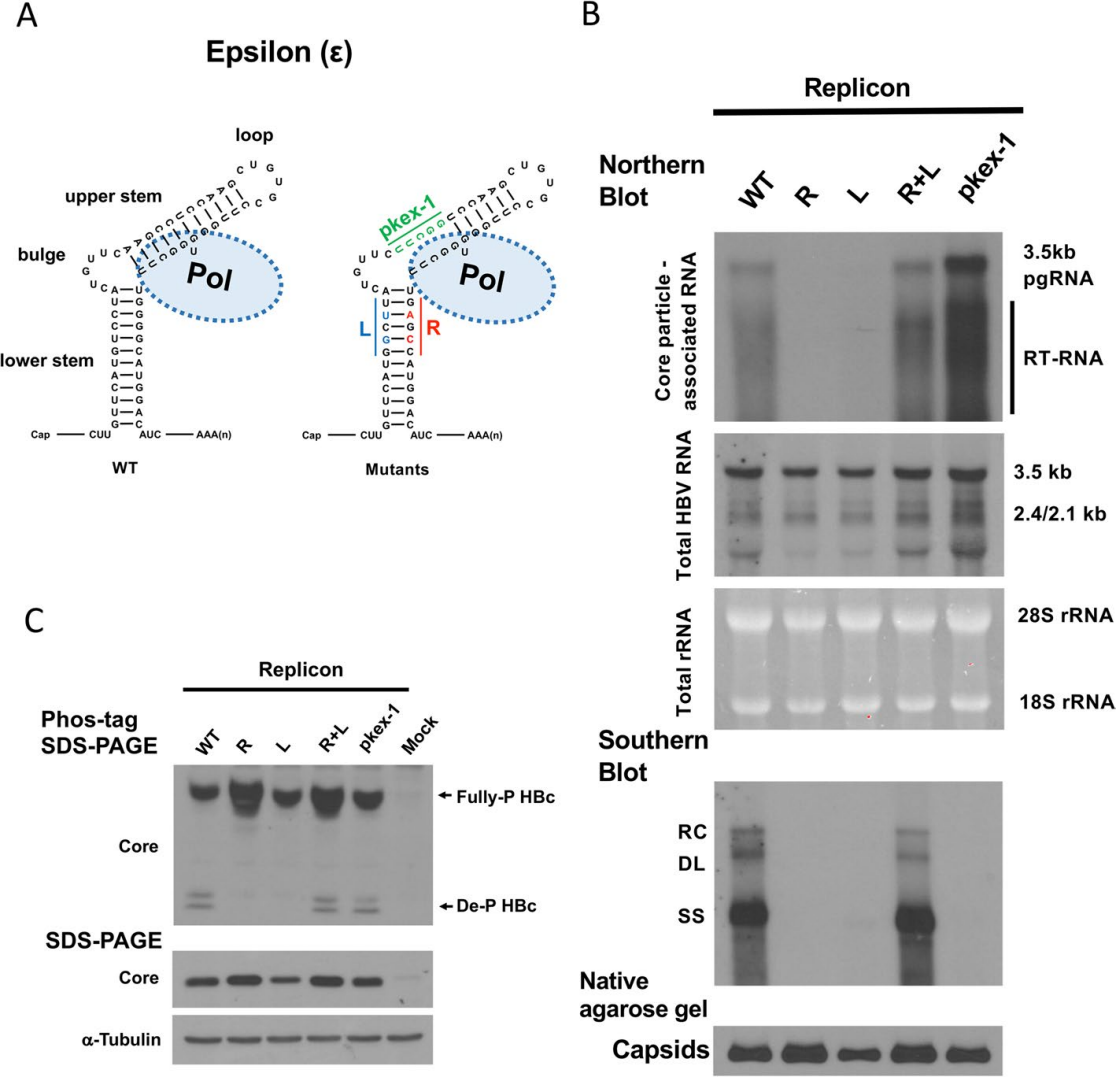
Dephosphorylation of HBc core protein is dependent on the encapsidation signal of the pregenomic RNA.** A** Encapsidation of pregenomic RNA (pgRNA) requires a highly ordered structure of the RNA packaging signal (epsilon) and polymerase. Epsilon mutant R and mutant L each contain a disrupted stem-loop structure of epsilon [68]. Double mutant R + L restored the wild type-like stem-loop structure of epsilon. Mutant pkex-1 contains a disrupted base pairing in the lower portion of the upper stem loop structure of the epsilon RNA [14]. **B** Upper panel: Single mutant R and single mutant L are defective in pgRNA encapsidation by Northern blot analysis, while the double mutant R + L restored pgRNA encapsidation. Mutant pkex-1 exhibited stronger signal intensity of encapsidated pgRNA than WT-HBV. Middle panel: Total cytoplasmic viral RNAs by Northern blot analysis. Lower panels: Viral DNA synthesis was detected only in WT-HBV and mutant R + L. RC: relaxed circle; DL: double-strand linear; SS: single-strand DNA

HBV polymerase is best known for its function in pgRNA encapsidation and viral DNA synthesis [53]. The former depends on the polymerase recognition of epsilon - a cis-element with a higher order stem-loop structure near the 5’end of pgRNA [3, 14, 21, 25, 72]. Since polymerase can affect HBc de-P (Fig. 1C), we examined in Fig. 2 whether the epsilon signal and pgRNA encapsidation could also affect HBc de-P. Unlike the wild type HBV, both the single epsilon mutant R and its compensatory mutant L lost the normal structure at the lower stem of epsilon [72] (Fig. 2A). Both mutants lost pgRNA encapsidation (Fig. 2B) and HBc de-P simultaneously (Fig. 2C). However, when the normal stem-loop structure of epsilon was restored in the double mutant R + L (Fig. 2A), both pgRNA encapsidation and DNA synthesis were almost completely recovered (Fig. 2B), and HBc de-P was rescued simultaneously by the Phos-tag gel assay (Fig. 2C). These results strongly suggest that core de-P is not only dependent on the polymerase (Fig. 1), but also hinges on the epsilon RNA structure and pgRNA encapsidation (Fig. 2). Since the polymerase is known to bind to the epsilon [3, 14, 21, 25, 72], most likely, it is the pol-epsilon complex that triggers HBc de-P.

### Uncoupling HBc de-P and replication

Previously, another epsilon mutant pkex-1 was engineered to contain a disrupted upper stem structure of the epsilon RNA [14]. This pkex-1 mutant exhibited strongly increased RNA encapsidation, no DNA synthesis (Fig. 2B), yet maintained core de-P in our Phos-tag gel assay (Fig. 2C). The dissociation between viral DNA synthesis and core de-P was examined in another experimental setting. When viral replication (SS and RC DNA synthesis) of wild type HBV was inhibited with therapeutic nucleos(t)ide analogs (Supplementary Figure S2A and S2B), we observed a strongly increased signal of de-P core protein (Supplementary Figure S2C). Taken together, core de-P is not sufficient for viral DNA synthesis and the timing of core de-P must occur before DNA synthesis, either during or immediately after pgRNA encapsidation [73].

### RNase H domain mutant and HBc de-P

**Fig. 3:**
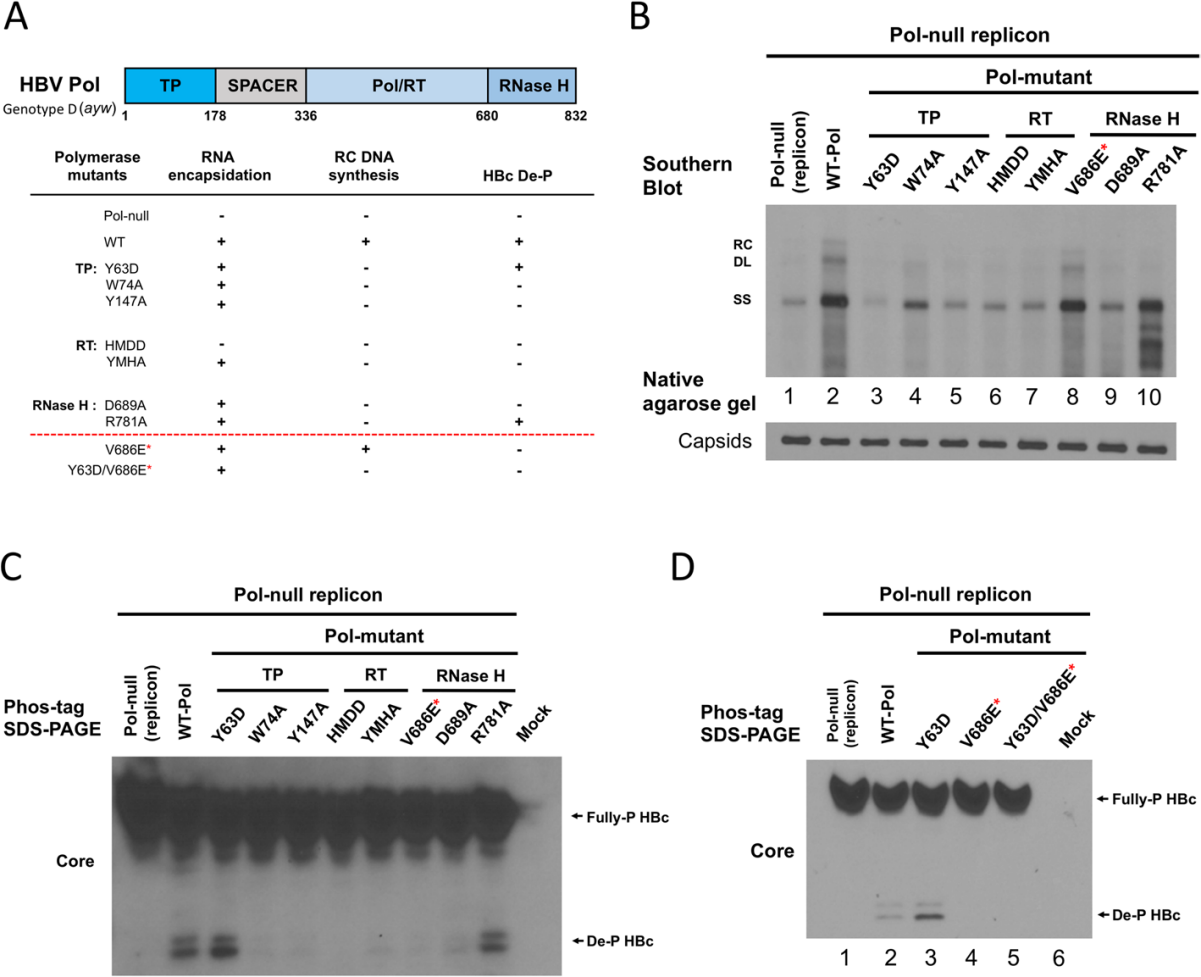
Dephosphorylation of HBc core protein is not required for viral DNA synthesis.** A** Different pol domain mutants were characterized for their viral DNA synthesis and HBc dephosphorylation. **B** HBV DNA synthesis can be detected in WT and mutant V686E (red asterisk) by Southern blot analysis. Due to the leaky polymerase expression in the CMV-driven replicon plasmid pol-null (Fig. 1; see Methods), trace amount of residual SS DNA can be detected after longer exposure of the X-ray film in the negative controls (lane 1 and 3). **C** A replication competent RNase H domain mutant V686E (red asterisk) exhibited no detectable dephosphorylated HBc by the Phos-tag gel. **D** No dephosphorylated HBc was detected in the double mutant Y63D/V686E (red asterisk)

HBV polymerase contains four different domains, including terminal protein (TP), spacer, reverse transcriptase (RT) and RNase H (Fig. 3A). Because of the association between polymerase and core de-P (Fig. 1), we set out to map the core de-P activity using a number of mutants in different polymerase domains. As shown in Fig. 3A, characterizations of eight different pol domain mutants were summarized here. These pol domain mutants were all designed according to the previous literature [9, 29, 33, 47, 54, 60, 61]. For example, similar to the replicon-only control experiment with no exogenous co-transfected polymerase (lane 1, Fig. 3B), mutant HMDD exhibited only a faint residual DNA signal after a longer exposure of the X-ray film in Southern blot. While the RC DNA is absent in most of the pol mutants, we noted that RNase H mutant R781A displayed a zebra-banding DNA pattern (lane 10, Fig. 3B).

### No need for HBc de-P in DNA synthesis

Surprisingly, one RNase H domain mutant V686E (red asterisk, Fig. 3B) was capable of synthesizing both SS and RC DNA replicative intermediates (Fig. 3B), often at a somewhat reduced level relative to the WT-HBV. This mutant V686E always exhibited no apparent core de-P (Fig. 3C). Therefore, in contrast to the previous concept extended from the phosphomicmicking mutagenesis experiments [16, 32], our combined results from the epsilon mutant pkex-1, the nucleo(s)tide analog treatment, and particularly the HBc de-P deficient mutant V686E, broke the tight correlation between core de-P and viral DNA synthesis. Similar to the epsilon mutant pkex-1, a polymerase TP domain mutant Y63D is known to accumulate a high level of encapsidated pgRNA, due to a defect in the initiation of viral DNA synthesis [9, 33] (Supplementary Figure S3A). This mutant Y63D exhibited a much stronger signal of de-P HBc than the WT on the Phos-tag gel (lane 3, Fig. 3D). However, the double mutant Y63D/V686E exhibited no detectable de-P HBc (lane 5, Fig. 3D), even though the amount of its encapsidated pgRNA was higher than the WT-HBV by Northern blot analysis (Supplementary Figure S3B). Therefore, as expected, core de-P is not required for pgRNA encapsidation. Altogether, these results of the double mutant Y63D/V686E indicated that the mutation V686E is dominant over the mutation Y63D in core de-P. Furthermore, the complete absence of the de-P HBc of mutant V686E, cannot be attributed to its somewhat attenuated viral replication.

### More RNase H domain mutants and HBc de-P

**Fig. 4:**
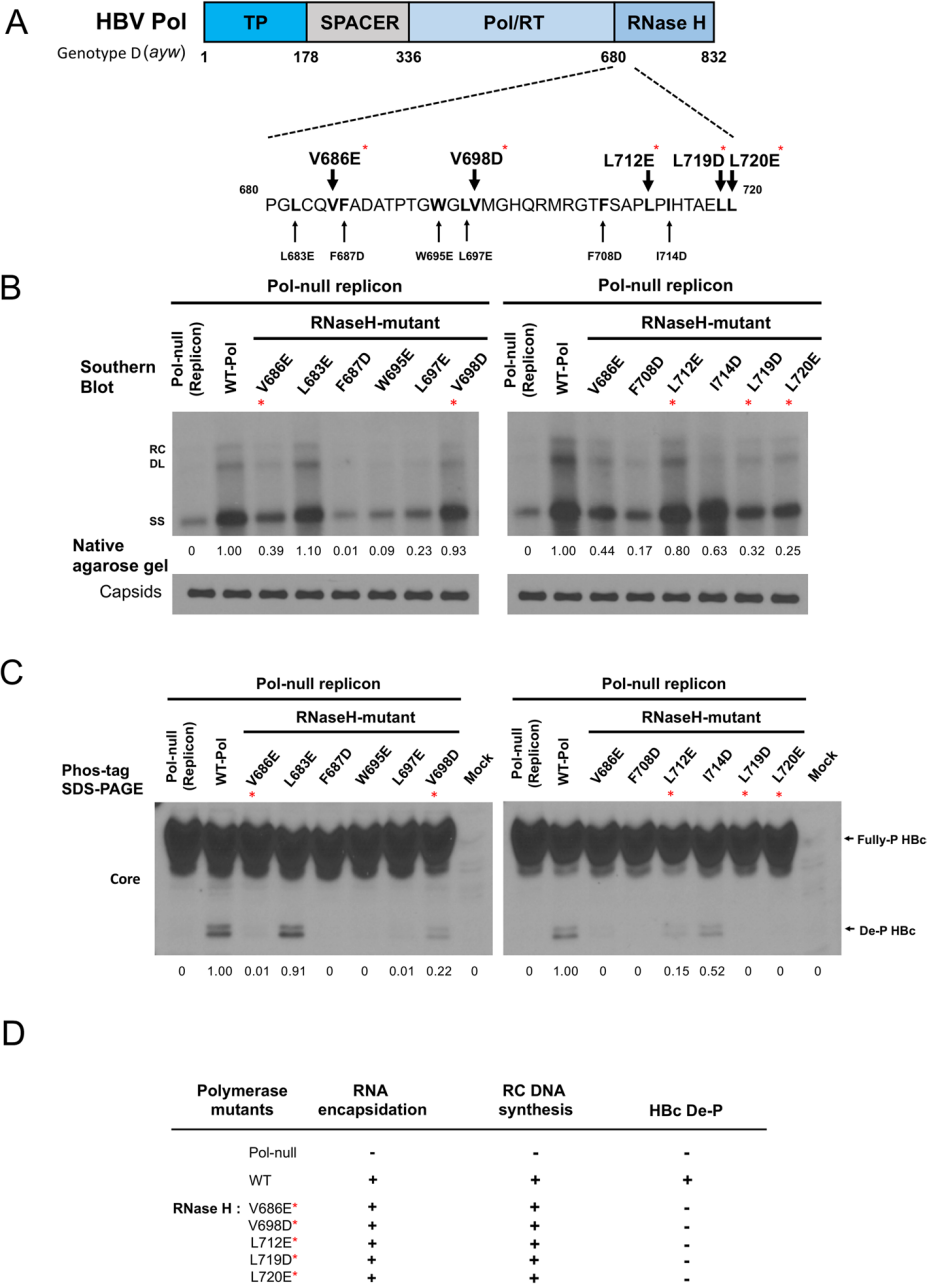
HBc dephosphorylation is neither necessary nor sufficient for viral DNA synthesis.** A** Eleven RNase H domain mutants were designed to contain substitutions from a hydrophobic to an acidic residue. **B** Viral DNA synthesis of these RNase H mutants was analyzed by Southern blot analysis. Red asterisks highlight mutants with dissociated activities of viral DNA synthesis in B) and HBc dephosphorylation in C). **C** Phos-tag gel analysis detected the presence or absence of dephosphorylated HBc protein. **D** A summary table of five RNase H domain mutants (red asterisk) which exhibited dissociation between HBc dephosphorylation and viral DNA synthesis

Next, we tested further if this mutation V686E is an idiosyncratic or a general phenomenon of RNase H domain mutants? By changing from hydrophobic to acidic residues in the RNase H domain between amino acid 680 and 720, we engineered a total of eleven site-directed substitution mutants in a polymerase expression vector pMT-pol (Fig. 4A). A pol-null replicon plasmid and various RNase H domain mutants in pMT-pol were cotransfected into HuH-7 cells for Southern blot and the Phos-tag gel assays. Like mutant V686E, mutants V698D, L712E, L719D, and L720E (red asterisk) are also replication competent (Fig. 4B), despite their nearly complete absence of de-P HBc (Fig. 4C). As summarized in Fig. 4D, five RNase H domain mutants, changing from a hydrophobic residue into an acidic residue, can undergo normal viral DNA synthesis without any significant HBc de-P. In contrast, changing from a hydrophobic residue into a neutral alanine or a basic arginine, did not clearly uncouple viral DNA synthesis and core de-P (Supplementary Figure S4).

### No need for HBc de-P in virion secretion

**Fig. 5:**
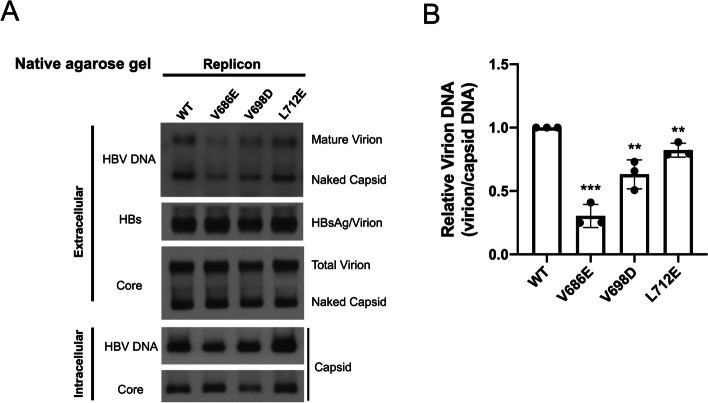
A moderate effect of HBc core dephosphorylation on genome-containing virion secretion.** A** Upper panel: Three putative phosphatase-deficient RNase H domain mutants secreted significantly less amount of virion-associated viral DNA than wild type HBV, while HBsAg/virions and naked capsids were similar between wild type and mutants. Middle panel: Extracellular viral and subviral particles were collected from the media of HuH-7 cells on day 5 post-transfection with WT-HBV and mutants. PEG precipitated particles were resolved by native agarose gel, followed by Western blot analyses using anti-core and anti-HBs antibodies. Lower panel: Intracellular capsid particles were analyzed for viral DNA and core by Southern and Western blot analyses. **B** A bar graph comparison of virion-associated viral DNAs between wild type and three RNase H domain mutants. Signal intensities of HBV DNA associated with mature virions in the top panel of A) were normalized to HBV DNAs associated with intracellular capsids at the bottom panel of A). Signal intensities were quantified by densitometry and an Image J software, *** *p* < 0.001, ** *p* < 0.01. p value refers to the Student’s t-test statistical analysis

A previous classic study revealed that the virion-associated core protein was highly increased in dephosphorylation, suggesting that a hypo-P core particle could be a prerequisite for virion secretion [44]. We examined virion secretion of HBc de-P-deficient mutants V686E, V698D and L712E by native agarose gel and Southern blot analysis (Fig. 5A). All three mutants exhibited only slightly reduced amount of mature genome-containing virions by approximately 2-fold or less, while their levels of secreted HBsAg, naked capsids, and virion-associated core protein were all similar to those of the WT-HBV control (top panel, Fig. 5A and B). It is well known that HBV empty virions exist in large excess to the DNA genome-containing virions in patients and cell culture [17, 41, 48]. Therefore, similar intensities of the core protein signal in total virions on the native agarose gel (middle panel, Fig. 5A) strongly suggested that the secretion of empty virions of mutants V686E, V698D and L712E were not appreciably affected by their absence of core de-P. Altogether, these results do not argue for the notion that core de-P is required for secretion of either empty or genome-containing virions. It is noteworthy that the level of secreted mature virions in Fig. 5A appears to be correlated with the level of de-P HBc in Fig. 4C. Therefore, even though HBc de-P is not indispensable for virion secretion, it remains possible that core de-P might still facilitate virion secretion.

### Need HBc de-P for viral infectivity

**Fig. 6:**
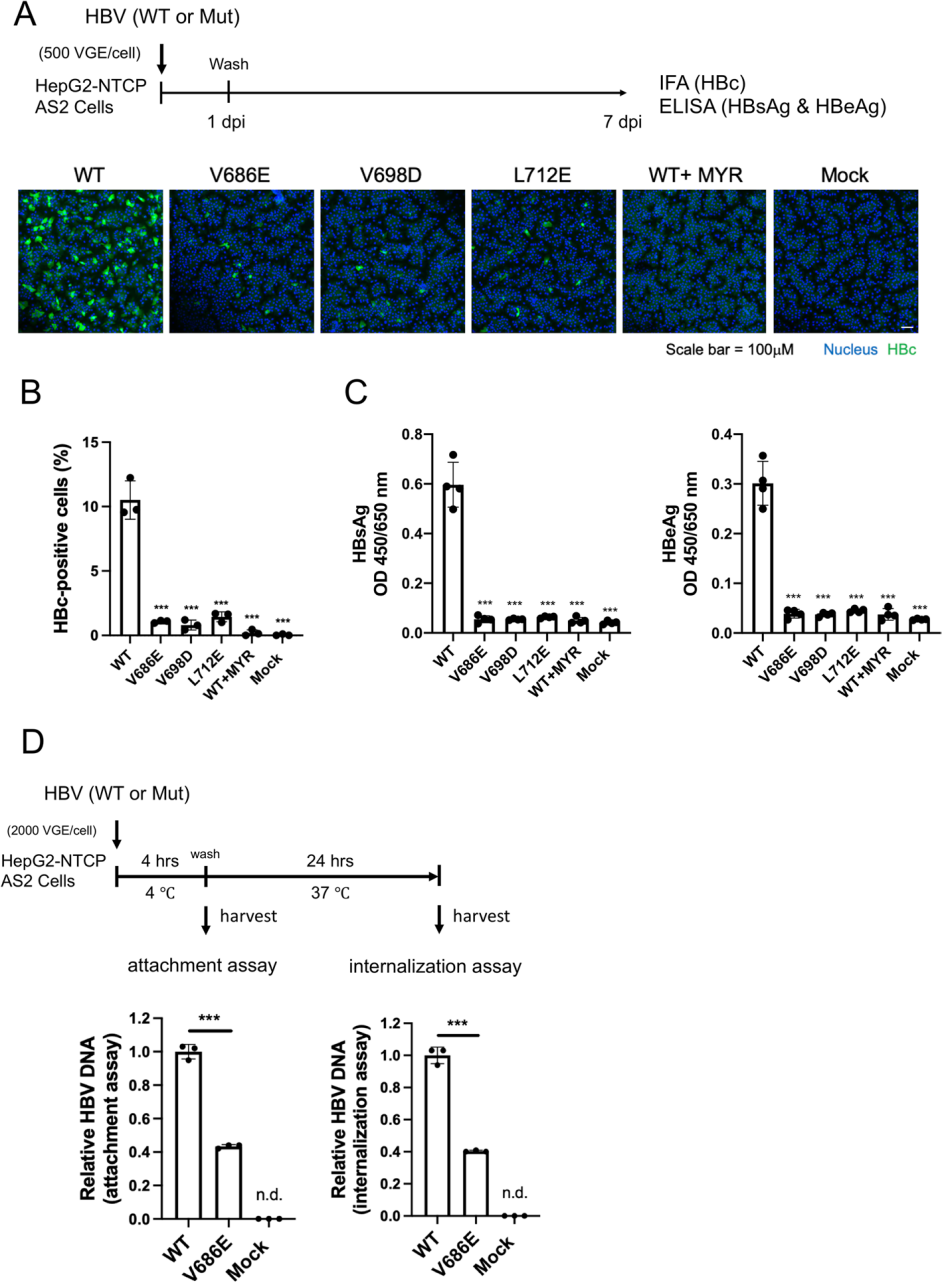
Core dephosphorylation-deficient RNase H mutants exhibited a 10-fold reduction in viral infectivity. **A** & (**B**) A 10-fold reduction in HBc core protein signal was detected by confocal IFA in RNase H  domain mutants deficient in putative phosphatase activity at 7 dpi. None of the pol mutation sites overlaps with the S envelope ORF. HepG2-NTCP-AS2 cells were in vitro infected with equal amounts of virions from WT-HBV and mutants defective in core dephosphorylation. MYR: a preS1 inhibitory control peptide. **C** The ELISA assays for HBsAg (left) and HBeAg (right) in the media detected a 10-fold reduction in cells infected with RNase H domain mutants. **D** RNase H domain mutant V686E exhibited a 2.5-fold reduction in attachment (left) and subsequent internalization (right). Naked capsids do not bind to NTCP and are present in near equal proportion in WT and mutant virions. HBV DNA was measured by qPCR. *** *p* < 0.001, *p* value refers to the Student’s t-test statistical analysis

**Fig. 7:**
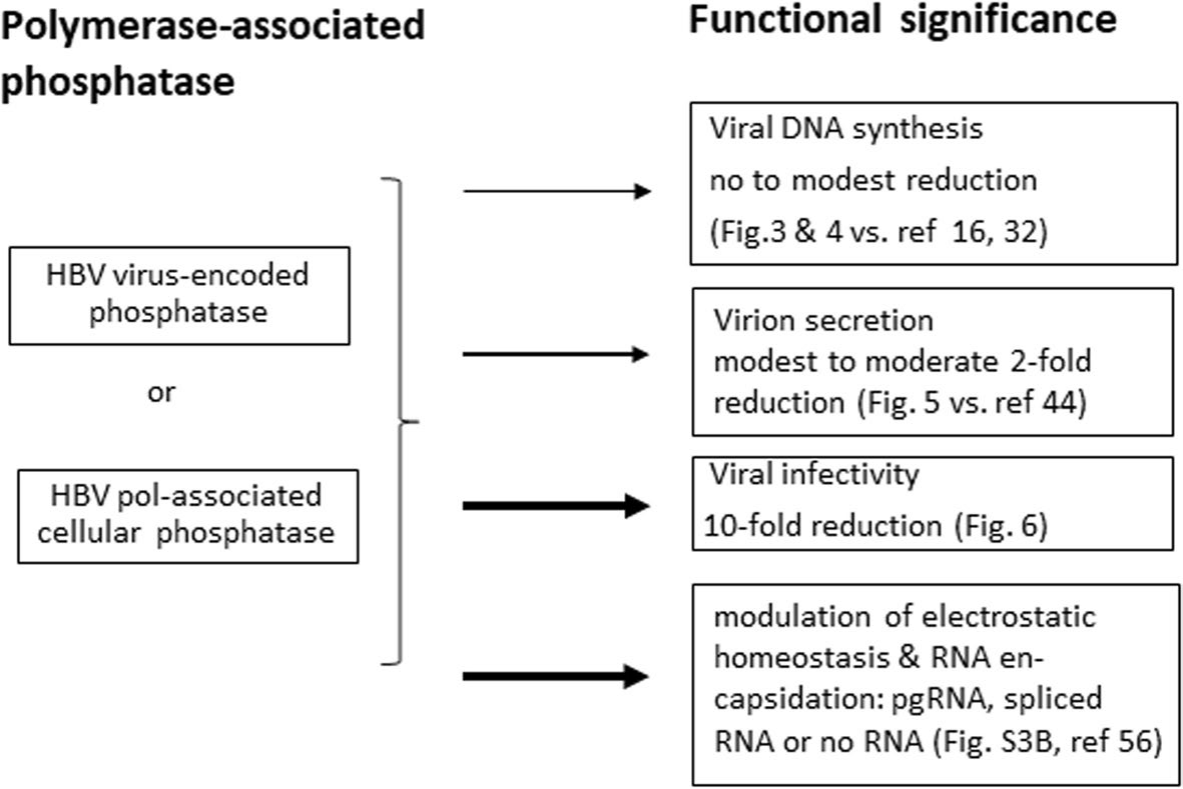
A summary of the functional significance of HBc de-phosphorylation  A novel putative phosphatase with an HBc de-P activity was detected at the C-terminal domain of HBV polymerase by site-directed mutagenesis and the Phos-tag gel assay. Depending on the specific position of the missense point mutation, the phosphatase-defective pol mutants exhibited various degrees of reduction in viral replication, virion secretion and viral infectivity. The origin of this putative phosphatase could come from an unknown intrinsic phosphatase encoded by the HBV pol ORF. Alternatively, it could come from a cellular phosphatase physically associated with HBV polymerase. In one of our previous reports [56], hyperphosphorylated capsids are biased to preferentially encapsidate shorter spliced RNAs, instead of the full-length 3.5 kb pgRNA. This putative phosphatase could play a regulatory role in pgRNA packaging and capsid assembly through modulating HBc dephosphorylation and thus maintaining electrostatic homeostasis in the capsid interior. Relative to the parental single mutant Y63D, core-particle-associated viral RNA is significantly reduced in the double mutant Y63D/V686E (Fig. S3B). This result suggests that the loss of HBc de-P by V686E can result in a reduced amount of viral RNA encapsidation. The thickness of arrows reflect their respective degree of functional effects upon the loss of HBc de-P

Since core de-P is not a prerequisite for viral replication and virion secretion, what could then be the biological significance of core de-P, if any? We compared the in vitro infectivity between WT-HBV and the HBc de-P-deficient mutants (Fig. 6; Methods). Equal amounts of genomic equivalent (GE) of WT and mutant virions were allowed to infect HepG2-NTCP-AS2 cells overnight, and confocal immunofluorescence assay (IFA) was performed to visualize core protein-positive cells at 7 dpi. Surprisingly, in sharp contrast to WT, the frequency of core-positive cells was significantly reduced by ~ 10 fold in three out of three mutants (Fig. 6A and B). Furthermore, HBsAg and HBeAg in the medium in all three mutants dropped to an almost undetectable level by ELISA at 7 dpi (Fig. 6C). We also performed the virus attachment assay by monitoring the binding of genome-containing virions to the HepG2-NTCP-AS2 cells at 4 °C for 4 h (left panel, Fig. 6D). In the subsequent assay for virus internalization, we switched the temperature from 4 °C to 37 °C for another 20 h post-binding (right panel, Fig. 6D). By qPCR analysis for HBV DNA, mutant V686E exhibited a significant reduction by 2.5-fold in both attachment and internalization assays. Therefore, loss of core de-P could compromise the efficiency of viral entry, which contributed to the lost infectivity. A summary of results is shown in Fig. 7.

## Discussion

Reverse transcriptase and RNase H were first found in retroviruses [1, 2, 55, 62]. Although HBV has a DNA genome, it replicates via an RNA intermediate [59]. Pregenomic RNA needs to be encapsidated before the initiation of reverse transcription [14]. Based on the phosphomimicking studies, phosphorylation at HBc ser-162 and ser-170 is required for HBV pgRNA encapsidation [16, 31, 32], but the EEE or DDD mutant (S155D/E, S162D/E and S170D/E) is severely defective in viral DNA synthesis [24, 31, 32, 35]. It is generally assumed that the lack of viral DNA synthesis of mutants DDD or EEE is caused by their irreversible lack of HBc de-P. Indeed, viral DNA synthesis appears to be well correlated with capsid de-P by the Phos-tag gel assay [56].

The polymerase TP domain mutant Y63D can encapsidate pgRNA (Supplementary Figure S3), but it is defective in the priming of reverse transcription [9, 33] (Fig. 3A and B). By Phos-tag gel analysis, mutant Y63D exhibited a strong signal of capsid de-P (Fig. 3C and D). Single mutant V686E is competent in DNA synthesis, yet defective in capsid de-P (Fig. 3B and C). In the context of a double mutant Y63D/V686E, mutation V686E still abrogated capsid de-P (Fig. 3D). Therefore, mutation V686E could inactivate capsid de-P either with or without viral DNA synthesis. Since there is no association between capsid de-P and viral DNA synthesis, our current study excluded a proposed essential role of HBc de-P in viral DNA synthesis (Figs. 3 and 4) [16, 32]. (see further discussion on electrostatic homeostasis below).

So far, we found that only the polymerase amino acid 680–720 (within the RNase H ORF) is associated with the HBc de-P. In theory, the de-P activity could originate from a cellular phosphatase complex associated with the RNase H domain of the viral polymerase. In this scenario, our HBc de-P-deficient mutants probably have lost their ability to bind to a putative cellular phosphatase. In literature, cellular protein phosphatase 1 (PP1) and phosphatase 2 A had been proposed to regulate HBc de-P [22, 65]. PP1 is known to dephosphorylate the majority of Ser/Thr-linked phosphorylations in eukaryotes [20]. Therefore, knockdown or overexpression of PP1 has a pleiotropic and global effect on many cellular machineries directly or indirectly, such as RNA polymerase II transcriptional termination [43] and eIF2α-mediated translational initiation [46]. In our co-immunoprecipitation experiment, Flag-PP1α appeared to bind to HA-Pol better than Flag-PP1β and Flag-PP1γ (Supplementary Figure S5A). However, in another experiment, we detected no apparent difference in the binding to Flag-PP1α or Flag-PP1β between HA-pol, HA-Pol-V686E and HA-Pol-V698D (Supplementary Figure S5B). There is no apparent correlation between HBc de-P and pol binding to the PP1 phosphatase.

In addition to the cellular phosphatase scenario, an alternative possibility is that a previously unidentified intrinsic phosphatase activity is directly encoded in the known RNase H open reading frame. Our experimental results from mutant V686E and mutant R781A strongly suggest that the RNase H activity and the HBc phosphatase activity are clearly distinct from each other. Mutant V686E is replication competent (lane 8, Fig. 3B) without any de-P HBc (Fig. 3C). Conversely, mutant R781 is RNase H-defective (zebra-banding of lane 10, Fig. 3B), yet fully competent for HBc de-P (next to the last lane, Fig. 3C). Importantly, it should be noted here that none of the HBc de-P-deficient mutants, such as V686E, V698D, L712E, L719D, and L720E, are defective in the replication and RNase H activity (Fig. 4).

It remains to be investigated whether HBV polymerase per se contains any HBc phosphatase activity. However, in our cotransfection experiment using pMT-pol and HBc expression plasmids, we detected no de-P HBc by the Phos-tag gel assay (Chih-Hsu Chang and Chiaho Shih, unpublished results). This result, by its negative nature, cannot be interpreted as the lack of a phosphatase activity intrinsic to HBV polymerase. In literature, both Streptomyces and Mycobacteria contain a bifunctional enzyme consisting of an RNase H domain and an acid phosphatase domain [23, 42]. It is unclear what could be the biological or evolutionary significance for a bacterial enzyme with dual functions in phosphatase and ribonuclease H. We are not aware of any viral polymerase or cellular telomerase with bifunctional activities in reverse transcriptase/RNase H and phosphatase.

It is generally believed that core de-P has a role in virus maturation and virion secretion. In our current study, several phosphatase defective mutants lack of core de-P, secreted a normal level of empty virions as well as a 2-fold reduced level of genome-containing virions. By using an infection assay, we observed a striking difference in viral infectivity between the wild type virus and the phosphatase deficient mutants. Viral attachment and internalization were also reduced by about 2.5-fold for these phosphatase mutants. As a side note, HBc ARD was shown to exhibit RNA chaperon activity in vitro and ARD phosphorylation dampened the RNA chaperon activity [10]. We detected only a mild to moderate degree of reduction in viral DNA synthesis in these de-P-defective mutants (Figs. 3 and 4). Therefore, in vitro RNA chaperon activity may not play a major role in HBV life cycle in vivo.

Nuclear transport of HBc could be influenced by its phosphorylation and dephosphorylation [27, 37]. In addition to viral entry, the decreased infectivity of these phosphatase mutants could also be caused by the post-entry events, such as a compromised nuclear import of the hyper-P capsids (Er-Yi Huang, Hung-Cheng Li, and Chiaho Shih, unpublished results). It is generally believed that mature capsids in the cytoplasm could directly bud into ER/Golgi for virion secretion [6]. Recently, we demonstrated that mature capsids in the cytoplasm need to be first transported into the nucleus in order to access the ER/Golgi compartment via the CRM1-mediated nuclear export pathway [57]. The potential significance of exosomes in virion secretion in a recent report also remains to be further investigated [64]. In our current study, core de-P appears to be required for embarking a new round of productive infection with the progeny viruses.

In our previous cryoEM studies of the full-length core particles, we observed filamentous density of ~ 20 Å in length protruding from the hole at each local three-fold position [71]. We interpret this filamentous density as part of the externally exposed HBc ARD tail. Our cryoEM observation of the exposed ARDs could explain the known ARD’s accessibility to protease digestion and antibody recognition [15, 49]. Here, we speculate that the HBc ARD phosphorylation status could influence its exposure to the capsid exterior [71], which in turn could affect multiple events pleiotropically. For example, ARD phosphorylation could influence its interactions with the cellular importin machinery during nuclear import of capsids, with the envelopment machinery during virion morphogenesis, the topological switch of the pre-S1 polypeptide to the virion surface [50], and ultimately the NTCP receptor binding. It is reminiscent that a phosphoserine at the capsid protein position 259 is required for initiation of DHBV infection [70].

Finally, it is tempting to speculate that this HBc de-P activity could contribute to the electrostatic homeostasis in the capsid interior during RNA encapsidation [11, 34, 39, 56]. For example, HBV is known to produce shorter spliced RNAs [7, 58]. In previous studies, highly phosphorylated capsids, with an excessive negative charge content, tend to encapsidate spliced shorter RNAs or even no RNA (empty capsids) [11, 34, 39, 56]. Here, consistent with the conceptual framework of electrostatic homeostasis, we noted that the double mutant Y63D/V686E contained no de-P HBc (i.e., increased negative charge content). To achieve charge balance, mutant Y63D/V686E appeared to package a significantly reduced amount of viral RNA than its parental mutant Y63D (Fig. 4D; Supplementary Figure S3B).

There are two interesting and very consistent observations in all of the Phos-tag gel results (Figs. 1C, 3 and 4; Supplementary Figure S2C and S4B). The first observation is the “bipolar pattern” of HBc phosphorylation on the Phos-tag gel. HBc ARD contains a total of 8 potential phosphorylation sites (*n* = 8) (Fig. 1A). The last serine-181 of HBc is not phosphorylated by SRPK1 in an *E. coli* co-expression system [19]. This bipolar pattern exhibits the slow-migrating hyper-P HBc (say *n* = 6, 7) and the frontier-running de-P doublet (*n* = 0, 1). We have never observed any HBc Phos-tag gel pattern with an intermediate degree of phosphorylation (say *n* = 3, 4). The second intriguing observation is the consistent association between pgRNA encapsidation and a characteristic de-P doublet near the bottom of every Phos-tag gel in this paper. This doublet probably represents completely de-P and mono-P HBc. If so, the exact location of the mono-P site can be mapped in further details, e.g., by using phosphorylation state and site specific antibodies or by mass spectrometry-based sequencing.

We provided here an explanation for the bipolar HBc Phos-tag gel pattern based on the electrostatic homeostasis concept [11, 34, 39, 56]. We postulate that capsids with intermediate-P (*n* = 3 or 4) may be in an “electrostatic dilemma” for capsid assembly and stability. On the one hand, to form a stable empty capsid without any encapsidated RNA,  the total negative charge content contributed from the intermediate-P HBc, may not have sufficient amount of negative charge to counter-balance the excessive amount of positive charged arginines of ARD. On the other hand, to form a stable RNA-containing capsid,  an excessive amount of negative charge in the capsid interior could be a problem. An imbalanced charge content, either positive or negative charge in excess, is not favorable for capsid assembly and stability. In brief, the bipolar pattern could be related to an electrostatic dilemma in charge balance and capsid assembly for the intermediate-P HBc (*n*=3, 4).

As for the front-running doublet on the HBc Phos-tag gel (Figs. 1C, 3 and 4; Supplementary Figures S2C and S4B), the upper band of the doublet probably represents a mono-P HBc and the lower band represents the HBc devoid of any phosphorylation. The ratio of signal intensities between the upper and lower bands of the doublet is around 1:2, indicating that de-P HBc is approximately 2-fold over the mono-P HBc. It is well known that the de-P HBc from the AAA mutant (with S-to-A mutations at HBc aa 155, 162, 170), cannot support pgRNA encapsidation [16, 31, 32]. Therefore, it is tempting to speculate that the optimal charge content for capsids to accommodate the 3.5 kb pgRNA could be those mosaic capsids, consisting of ~ 33% mono-P and ~ 66% de-P HBc. In other words, for productive pgRNA encapsidation and assembly of an icosahedral capsid (T = 4, 240-mer), the most favored stoichiometry for pgRNA encapsidation could be a mixture of ~ 80 copies of mono-P and ~ 160 copies of de-P HBc molecules. It remains unclear whether a pol-associated phosphatase could also be found in other systems, such as HIV, the telomerase complex, or retrotransposons.

## Conclusion

It is a totally unexpected finding that the C-terminal domain of the polymerase is associated with a novel phosphatase activity responsible for HBc de-P. In contrast to the conventional wisdom, HBc de-P is not required for viral DNA synthesis and virion secretion. This putative polymerase-associated phosphatase appears to play a functional role in modulating the electrostatic homeostasis in capsid interior and is crucial for viral infectivity (Fig. 7).

### Supplementary Information

**Supplementary Material 1.**

## Data Availability

All data generated in this study are included in this manuscript.

## Abbreviations

HBV: Human hepatitis B virus (HBV)
DHBV: Duck hepatitis B virus
WT: Wild type
HBc: HBV core protein
HBsAg: HBV surface antigen
RC DNA: Relaxed circle DNA
SS DNA: Single-stranded DNA
DS DNA: Double-stranded DNA
ARD: Arginine-rich domain
RNase H: An enzyme encodes a ribonuclease activity which degrades the RNA moiety of RNA-DNA hybrids
NAGE: Native agarose gel electrophoresis
