# Supplementary material for: Significance of hepatitis B virus capsid dephosphorylation via polymerase
Source: J Biomed Sci. 2024 Apr 1;31:34. doi: 10.1186/s12929-024-01022-9 (PMC10983652; doi:10.1186/s12929-024-01022-9)
Supplement: Supplementary file 1 — Supplementary Material 1. [file 12929_2024_1022_MOESM1_ESM.pptx]

## Slide 1
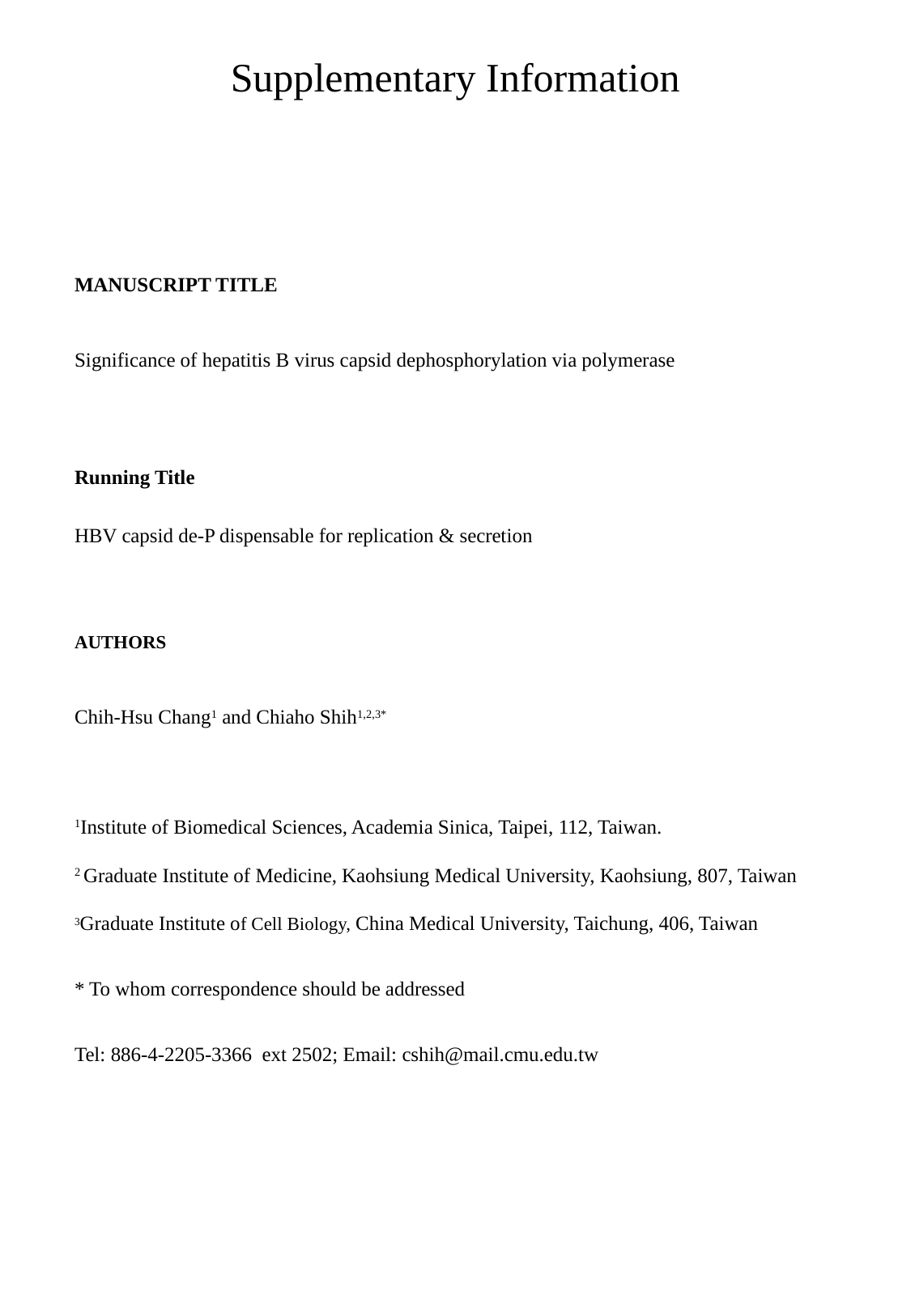

# Supplementary Information
MANUSCRIPT TITLE
Significance of hepatitis B virus capsid dephosphorylation via polymerase
Running Title
HBV capsid de-P dispensable for replication & secretion
AUTHORS
Chih-Hsu Chang1 and Chiaho Shih1,2,3*
1Institute of Biomedical Sciences, Academia Sinica, Taipei, 112, Taiwan.
2 Graduate Institute of Medicine, Kaohsiung Medical University, Kaohsiung, 807, Taiwan
3Graduate Institute of Cell Biology, China Medical University, Taichung, 406, Taiwan
* To whom correspondence should be addressed
Tel: 886-4-2205-3366 ext 2502; Email: cshih@mail.cmu.edu.tw

## Slide 2
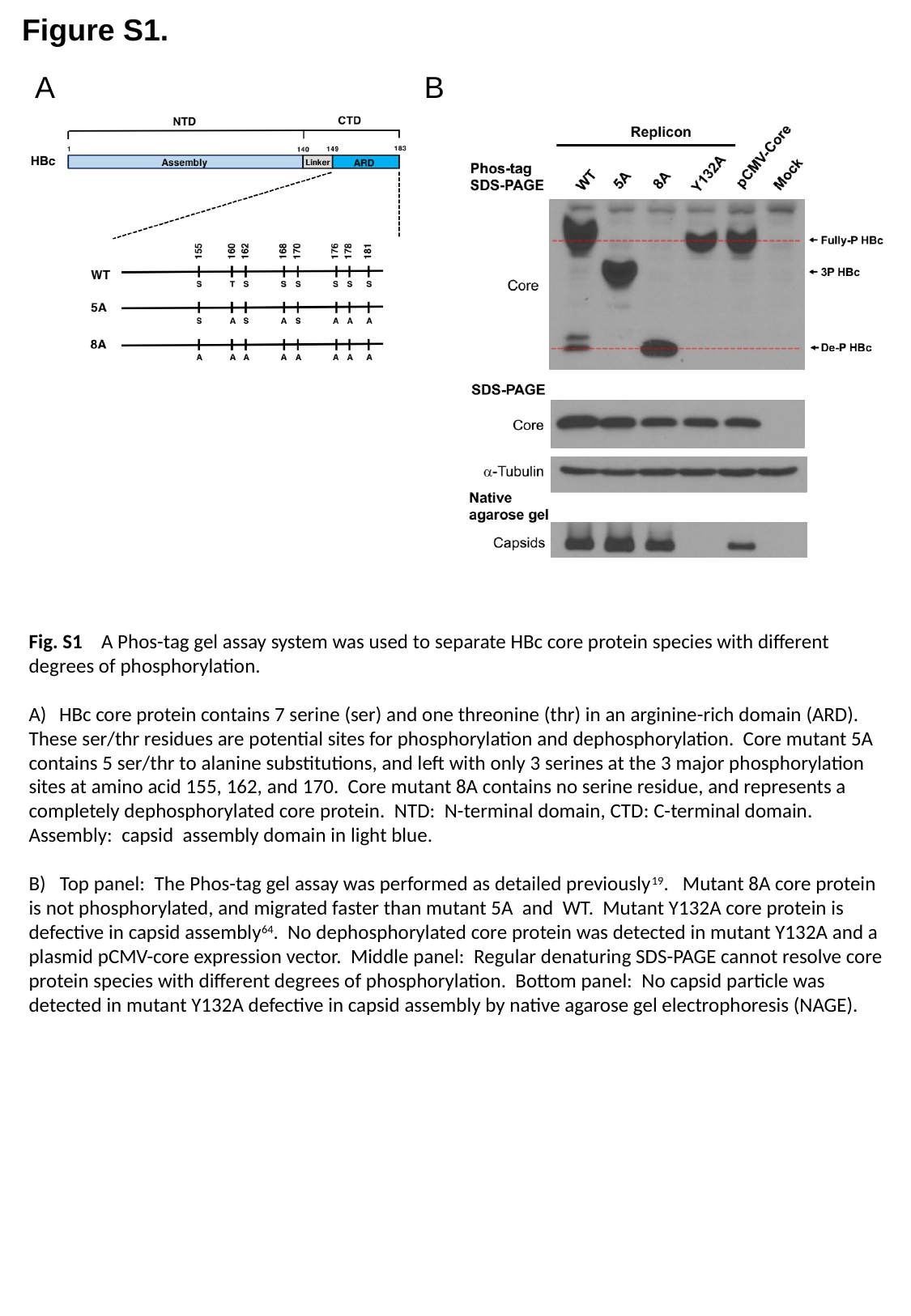

Figure S1.
A
B
Fig. S1 A Phos-tag gel assay system was used to separate HBc core protein species with different degrees of phosphorylation.
HBc core protein contains 7 serine (ser) and one threonine (thr) in an arginine-rich domain (ARD).
These ser/thr residues are potential sites for phosphorylation and dephosphorylation. Core mutant 5A
contains 5 ser/thr to alanine substitutions, and left with only 3 serines at the 3 major phosphorylation sites at amino acid 155, 162, and 170. Core mutant 8A contains no serine residue, and represents a completely dephosphorylated core protein. NTD: N-terminal domain, CTD: C-terminal domain. Assembly: capsid assembly domain in light blue.
 Top panel: The Phos-tag gel assay was performed as detailed previously19. Mutant 8A core protein is not phosphorylated, and migrated faster than mutant 5A and WT. Mutant Y132A core protein is defective in capsid assembly64. No dephosphorylated core protein was detected in mutant Y132A and a plasmid pCMV-core expression vector. Middle panel: Regular denaturing SDS-PAGE cannot resolve core protein species with different degrees of phosphorylation. Bottom panel: No capsid particle was detected in mutant Y132A defective in capsid assembly by native agarose gel electrophoresis (NAGE).

## Slide 3
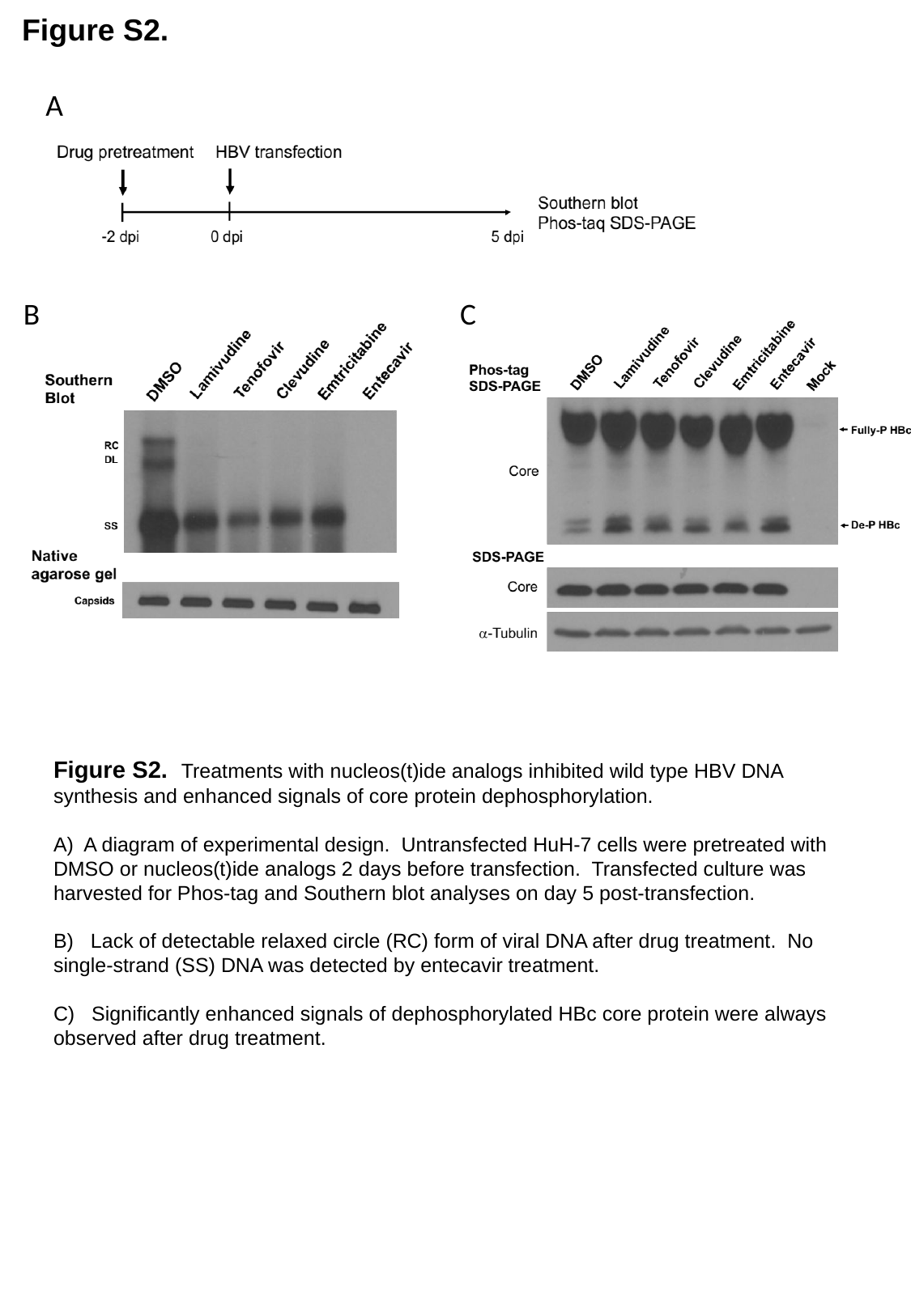

Figure S2.
A
B
C
Figure S2. Treatments with nucleos(t)ide analogs inhibited wild type HBV DNA synthesis and enhanced signals of core protein dephosphorylation.
A) A diagram of experimental design. Untransfected HuH-7 cells were pretreated with DMSO or nucleos(t)ide analogs 2 days before transfection. Transfected culture was harvested for Phos-tag and Southern blot analyses on day 5 post-transfection.
B) Lack of detectable relaxed circle (RC) form of viral DNA after drug treatment. No single-strand (SS) DNA was detected by entecavir treatment.
C) Significantly enhanced signals of dephosphorylated HBc core protein were always observed after drug treatment.

## Slide 4
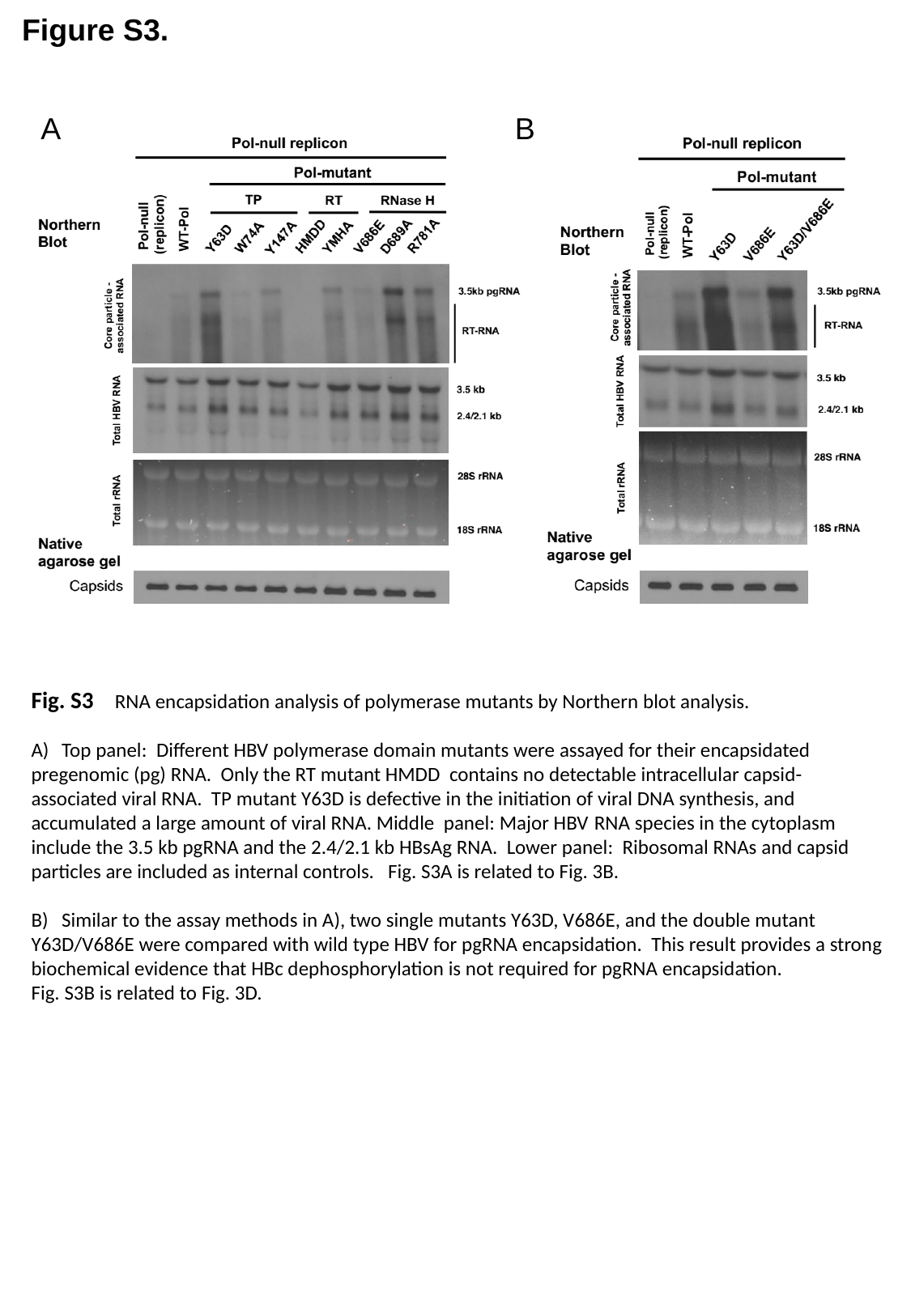

Figure S3.
A
B
Fig. S3 RNA encapsidation analysis of polymerase mutants by Northern blot analysis.
Top panel: Different HBV polymerase domain mutants were assayed for their encapsidated
pregenomic (pg) RNA. Only the RT mutant HMDD contains no detectable intracellular capsid-associated viral RNA. TP mutant Y63D is defective in the initiation of viral DNA synthesis, and accumulated a large amount of viral RNA. Middle panel: Major HBV RNA species in the cytoplasm include the 3.5 kb pgRNA and the 2.4/2.1 kb HBsAg RNA. Lower panel: Ribosomal RNAs and capsid particles are included as internal controls. Fig. S3A is related to Fig. 3B.
Similar to the assay methods in A), two single mutants Y63D, V686E, and the double mutant
Y63D/V686E were compared with wild type HBV for pgRNA encapsidation. This result provides a strong
biochemical evidence that HBc dephosphorylation is not required for pgRNA encapsidation.
Fig. S3B is related to Fig. 3D.

## Slide 5
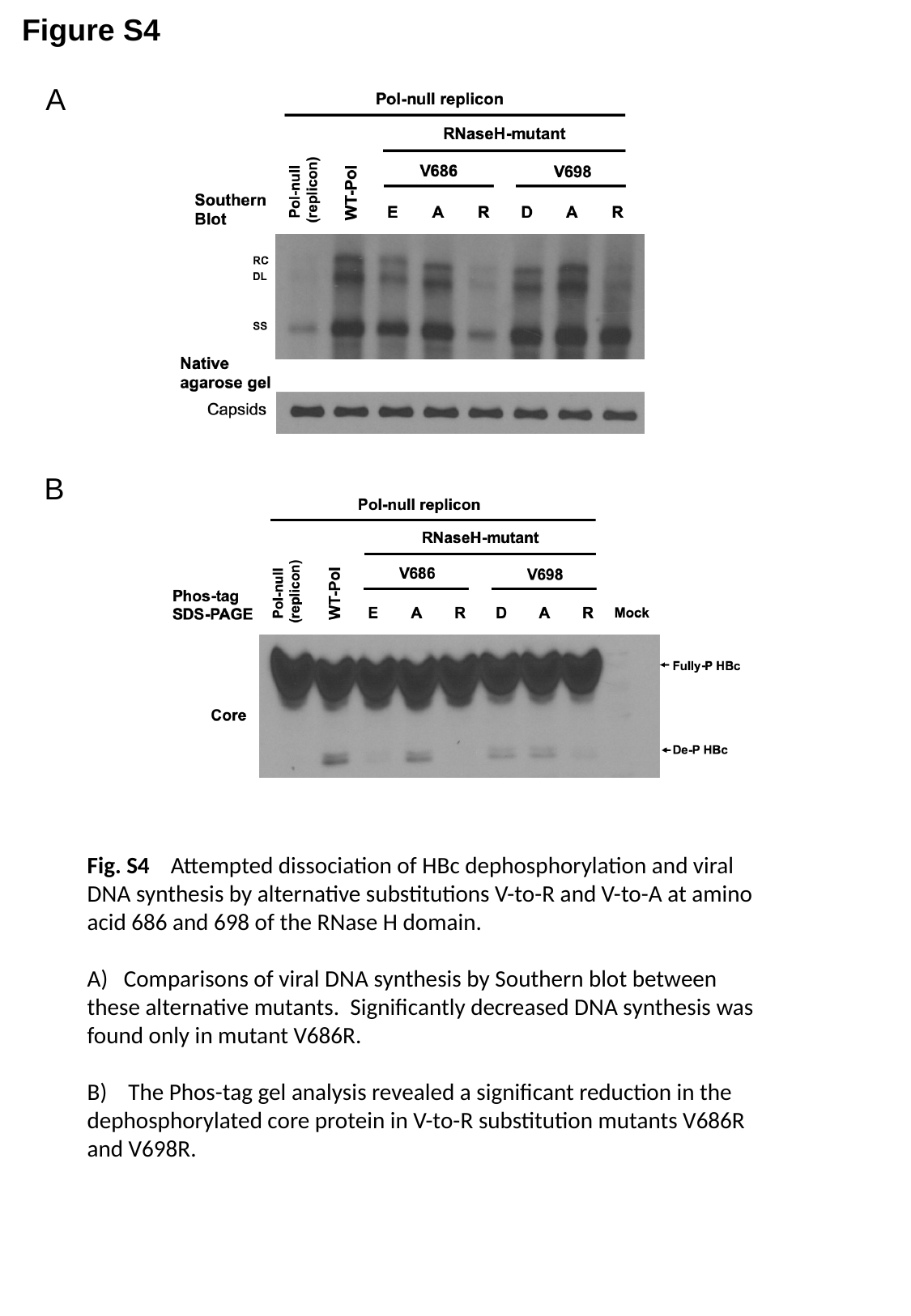

Figure S4
A
B
Fig. S4 Attempted dissociation of HBc dephosphorylation and viral DNA synthesis by alternative substitutions V-to-R and V-to-A at amino acid 686 and 698 of the RNase H domain.
A) Comparisons of viral DNA synthesis by Southern blot between these alternative mutants. Significantly decreased DNA synthesis was found only in mutant V686R.
B) The Phos-tag gel analysis revealed a significant reduction in the dephosphorylated core protein in V-to-R substitution mutants V686R and V698R.

## Slide 6
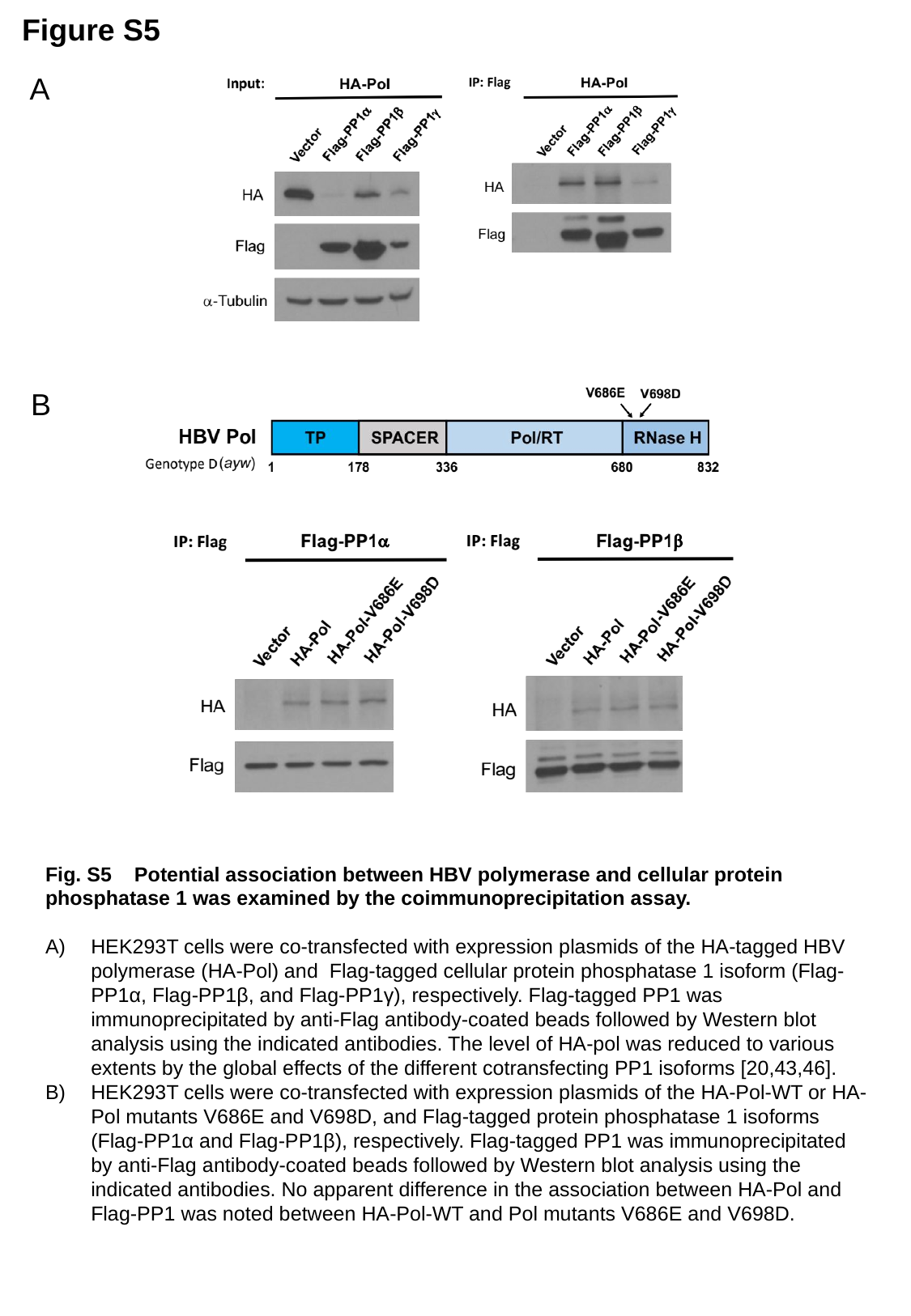

Figure S5
A
B
Fig. S5 Potential association between HBV polymerase and cellular protein phosphatase 1 was examined by the coimmunoprecipitation assay.
HEK293T cells were co-transfected with expression plasmids of the HA-tagged HBV polymerase (HA-Pol) and Flag-tagged cellular protein phosphatase 1 isoform (Flag-PP1α, Flag-PP1β, and Flag-PP1γ), respectively. Flag-tagged PP1 was immunoprecipitated by anti-Flag antibody-coated beads followed by Western blot analysis using the indicated antibodies. The level of HA-pol was reduced to various extents by the global effects of the different cotransfecting PP1 isoforms [20,43,46].
HEK293T cells were co-transfected with expression plasmids of the HA-Pol-WT or HA-Pol mutants V686E and V698D, and Flag-tagged protein phosphatase 1 isoforms (Flag-PP1α and Flag-PP1β), respectively. Flag-tagged PP1 was immunoprecipitated by anti-Flag antibody-coated beads followed by Western blot analysis using the indicated antibodies. No apparent difference in the association between HA-Pol and Flag-PP1 was noted between HA-Pol-WT and Pol mutants V686E and V698D.
